# *Scutellaria baicalensis* Alleviates Insulin Resistance in Diet-Induced Obese Mice by Modulating Inflammation

**DOI:** 10.3390/ijms20030727

**Published:** 2019-02-08

**Authors:** Hyun-Young Na, Byung-Cheol Lee

**Affiliations:** Department of Clinical Korean Medicine, Graduate School, Kyung Hee University, 26 Kyungheedae-ro, Dongdaemun-gu, Seoul 02447, Korea; hyun-512@hanmail.net

**Keywords:** *Scutellaria baicalensis*, insulin resistance, inflammation, adipose tissue macrophage, Kupffer cell

## Abstract

Insulin resistance is strongly associated with the metabolic syndrome, and chronic inflammation is known to be a major mechanism of insulin resistance and is a therapeutic target. This study was designed to evaluate the effect of *Scutellaria baicalensis* (SB) in high-fat diet (HFD)-induced insulin-resistant mice and to investigate its mechanism based on inflammatory responses. Mice were fed a HFD to induce insulin resistance and then administered SB for nine weeks. Body weight, glucose, lipid, insulin, epididymal fat pad and liver weights, and histologic characteristics were evaluated to determine the effect on insulin resistance. In order to evaluate the effects on the inflammatory process, we analyzed the proportions of macrophages in liver and epididymal fat and measured inflammatory gene expression. Fasting and postprandial glucose, fasting insulin, HOMA-IR, triglycerides, and low density lipoprotein cholesterol levels were significantly decreased by SB administration. The epididymal fat and liver showed significant weight decreases and histological improvements. Total adipose tissue macrophages (ATMs) decreased (27.71 ± 3.47% vs. 45.26 ± 7.26%, *p* < 0.05), M2 ATMs increased (47.02 ± 6.63% vs. 24.28 ± 8.00%, *p* < 0.05), and CD11b^+^ Kupffer cells decreased. The expression levels of tumor necrosis factor alpha and F4/80 in the liver were significantly decreased (12.03 ± 1.47% vs. 25.88 ± 4.57%, *p* < 0.05) compared to HFD group. These results suggest that SB improved insulin resistance through inhibition of macrophage-mediated inflammation.

## 1. Introduction

Metabolic syndrome represents a clustering of metabolic risk factors including central obesity, hyperinsulinemia, hypertension, and dyslipidemia; insulin resistance (IR) is considered a common mechanism underlying derangements associated with metabolic syndrome [1]. It is known that chronic, subacute inflammation plays an important role in the development and progression of insulin resistance. In particular, white adipose tissue plays an active role in this process by secreting inflammatory substances, including tumor necrosis factor alpha (TNF-α), interleukin 6, and monocyte chemoattractant protein-1 [2]. Inflammation in adipose tissue promotes the accumulation of macrophages and inflammatory cytokines produced by both adipocytes and macrophages, which causes systemic as well as local inflammation [2,3,4]. Similar inflammatory changes occur in the liver. Lipid accumulation in the liver is often accompanied by insulin resistance, which causes subacute hepatocellular inflammation and increases the release of pro-inflammatory cytokines [5]. These local pro-inflammatory substances, in addition to systemic inflammatory agents entering through the portal vein, activate Kupffer cells and other immune cells, which in turn release inflammatory cytokines and affect insulin resistance [4].

The root of *Scutellaria baicalensis* Georgi (SB) has been used for the treatment of fever, vomiting, dysentery, jaundice, respiratory infections, and skin diseases [6]. In several studies, SB showed favorable anti-inflammatory effects [7,8]; however, most studies investigated drug-induced acute inflammation. In terms of insulin resistance and related diseases, the effects of SB on obesity, hypertriglyceridemia [9], and hepatomegaly [10] have been reported, but there have been few related studies. Thus, in this study, we investigated the effects of SB on insulin resistance in high-fat diet (HFD)-fed C57BL/6 mice, and investigated the mechanisms of action with a focus on macrophage-mediated chronic inflammation.

## 2. Results

### 2.1. Effects of SB on BW and Epididymal Fat and Liver Weight Changes

**Figure 1:**
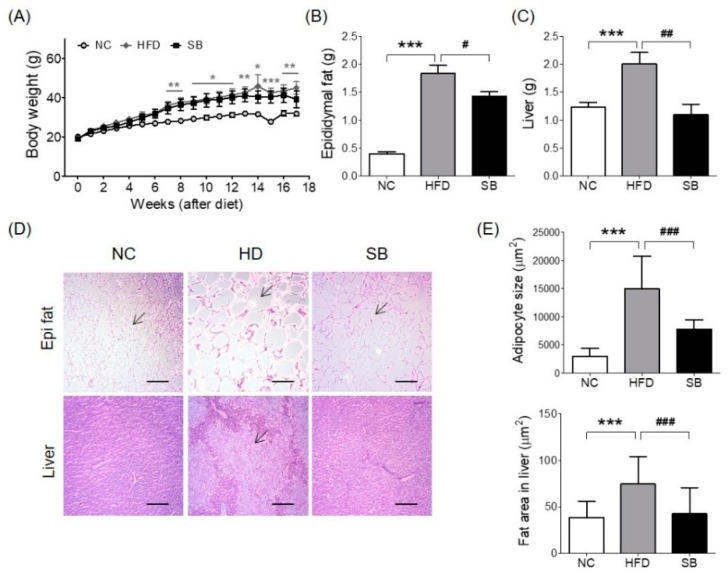
Effects of SB on body weight (**A**), epididymal fat (**B**), liver weight (**C**), histological changes in epididymal fat and liver. Representative histological images were assessed by hematoxylin and eosin (H&E) staining, scale bar indicates 100 μm, and arrow indicates adipocyte in Epi fat, 25 μm and arrow indicates fat deposition area in Liver (**D**), adipocyte size and fat area in liver (**E**). *n* = 6 in each group. Data shown as mean ± standard error of the mean (SEM). * *p* < 0.05, ** *p* < 0.01, *** *p* < 0.001, HFD compared with NC; # *p* < 0.05, ## *p* < 0.01, and ### *p* < 0.001, HFD compared with SB.

Body weight gain was higher in the high-fat diet (HFD) and SB groups than in the normal chow (NC) group, and the SB group had a lower body weight compared to the HFD group, although not significantly (39.14 ± 4.24 g vs. 44.98 ± 3.15 g) (Figure 1A). The epididymal fat pad weight was significantly higher in the HFD group compared to the NC group (*p* < 0.001), and the SB group had a significantly reduced epididymal fat pad weight compared to the HFD group (1.43 ± 0.08 g vs. 1.83 ± 0.15 g, *p* < 0.05) (Figure 1B). Adipocyte size showed a similar tendency. The adipocyte size of the HFD group was significantly greater than that of the NC group (*p* < 0.001), and that of the SB group was significantly lower compared to the HFD group (7795.02 ± 1679.15 μm^2^ vs. 14,941.78 ± 5815.63 μm^2^, respectively; *p* < 0.001) (Figure 1D,E). Liver weight was significantly higher in the HFD group than in the NC group (*p* < 0.001). However, in the SB group, liver weight was significantly lower compared to the HFD group (1.10 ± 0.19 g vs. 2.00 ± 0.21 g, respectively, *p* < 0.01) (Figure 1C). Liver fat area was also significantly higher in the HFD group compared to the NC group (*p* < 0.001), and in the SB group it was significantly lower than in the HFD group (42.55 ± 28.00 μm^2^ vs. 74.91 ± 29.15 μm^2^, *p* < 0.001) (Figure 1D,E).

### 2.2. Effects of SB on Insulin Resistance and Glucose and Lipid Metabolism

**Figure 2:**
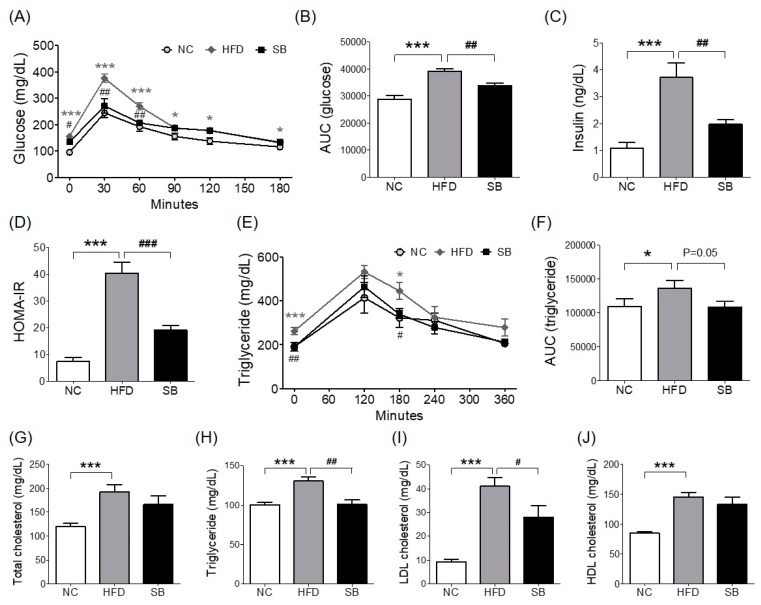
Effects of SB on oral glucose tolerance test (OGTT) and area under curve (AUC) (**A**,**B**), fasting insulin (**C**), HOMA-IR (**D**), oral fat tolerance test (OFTT) and area under curve (AUC) (**E**,**F**), serum total cholesterol, triglyceride, LDL cholesterol, and HDL cholesterol (**G**–**J**). *n* = 6 in each groups. Data shown as mean ± SEM. * *p* < 0.05, *** *p* < 0.001, HFD compared with NC; # *p* < 0.05, ## *p* < 0.01, and ### *p* < 0.001, HFD compared with SB.

To investigate insulin resistance, homeostatic model assessment for insulin resistance (HOMA-IR) was measured. HOMA-IR was significantly higher in the HFD group compared with both the NC and the SB groups (40.38 ± 3.99 vs 7.37 ± 1.49 and 19.02 ± 1.85, *p* < 0.001) (Figure 2D). Similarly, fasting insulin level was significantly lower in the SB group than in the HFD group (*p* < 0.01, Figure 2C). In the oral glucose tolerance test (OGTT), the blood glucose level in the HFD group was significantly higher at every time point than in the NC group, and the SB group had significantly lower blood glucose levels at 0, 30, and 60 min (0 min, *p* < 0.05; 30 min, *p* < 0.01; 60 min, *p* < 0.01) (Figure 2A). The HFD group had a significantly higher glucose area under curve (AUC) compared to the NC group (*p* < 0.001), and the SB group had a significantly lower AUC compared to the HFD group (33,801.00 ± 961.28 vs. 39,195.00 ± 899.06, *p* < 0.01) (Figure 2B).

For the lipid profile study, we measured total cholesterol (TC), triglyceride (TG), low density lipoprotein (LDL), and high density lipoprotein (HDL) cholesterol levels. In the HFD group, serum TC, TG, LDL, and HDL cholesterol levels were higher than in the NC group (*p* < 0.001). Compared to the HFD group, the SB group had significantly lower TG (101.00 ± 5.36 mg/dL vs. 130.33 ± 5.34 mg/dL, *p* < 0.01) and LDL cholesterol levels (28.00 ± 4.77 mg/dL vs. 41.00 ± 3.74 mg/dL, *p* < 0.05). The TC level in the SB group was lower than in the HFD group, but not significantly (165.60 ± 18.88 mg/dL vs. 191.83 ± 15.82 mg/dL), and similarly HDL-cholesterol in the SB group was lower than in the HFD group, but not significantly (144.85 ± 7.92 mg/dL vs. 132.58 ± 12.87 mg/dL) (Figure 2G–J). Oral fat tolerance test (OFTT) was also performed to evaluate the effect of SB on lipid metabolism. During the OFTT, the TG levels of all groups were highest at 120 min and then gradually decreased. The HFD group showed a higher TG level at every time point compared with the NC group, and the SB group showed a lower TG level at 0 min (*p* < 0.01) and 180 min (*p* < 0.05) compared to the HFD group (Figure 2E). The TG AUC was also significantly higher in the HFD group compared with the NC group, and the SB group had a lower TG AUC than the HFD group, but not significantly (108,366.00 ± 8507.87 vs. 136,335.00 ± 11476.50, p = 0.05) (Figure 2F).

### 2.3. Effects of SB on Adipose Tissue Macrophages (ATMs) and Liver Kupffer Cells

**Figure 3:**
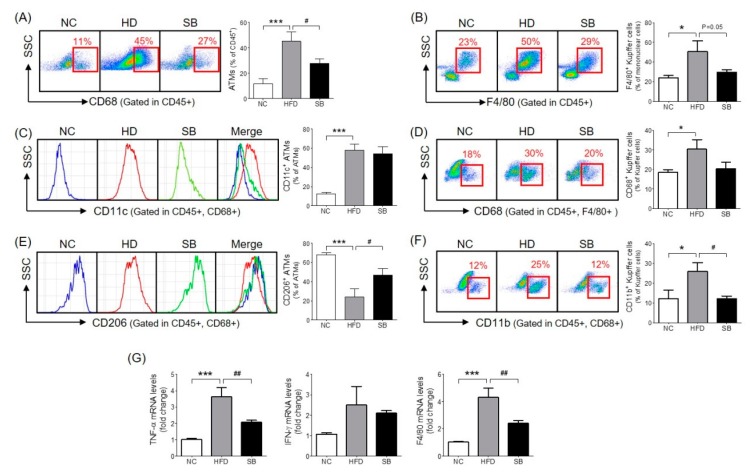
Effect of SB on ATM infiltration rate (**A**), CD206- and CD11c-positive ATMs (**C**,**E**), Kupffer cell infiltration rate (**B**), CD68- and CD11b-positive Kupffer cells (**D**,**F**) and inflammatory cytokine gene expression (**G**). *n* = 6 in each groups. Data shown as mean ± SEM. * *p* < 0.05, *** *p* < 0.001, HFD compared with NC; # *p* < 0.05, and ## *p* < 0.01, HFD compared with SB.

The ATM percentage was significantly higher in the HFD group compared to the NC group (*p* < 0.001), and the SB group had a significantly lower ATM percentage compared to the HFD group (27.71 ± 3.47% vs. 45.26 ± 7.26%, *p* < 0.05) (Figure 3A). In the analysis of the ATM subpopulations, the proportion of CD11c^+^ ATM (inflammatory macrophages) in the total ATM was significantly higher in the HFD group than in the NC group (57.77 ± 6.75% vs. 12.52 ± 1.54%, *p* < 0.001), but there was no significant difference between the SB and the HFD groups (54.11 ± 7.54% vs. 57.77 ± 6.75%) (Figure 3C). The percentage of CD206^+^ ATM (anti-inflammatory macrophages) in the total ATM was significantly lower in the HFD group compared to the NC group (*p* < 0.001), and was significantly higher in the SB group compared to the HFD group (47.02 ± 6.63% vs. 24.28 ± 8.00%, *p* < 0.05) (Figure 3E).

To investigate the effect of SB on liver immune cell composition, we measured the percentage of Kupffer cells among the total mononuclear cells and the percentages of CD68^+^ and CD11b^+^ Kupffer cells. The Kupffer cell percentage was significantly higher in the HFD group compared to the NC group (*p* < 0.05), and lower in the SB group compared to the HFD group (29.51 ± 2.77% vs. 50.77 ± 10.58%, *p* = 0.05) (Figure 3B). The ratio of phagocytic CD68^+^ Kupffer cells was also significantly higher in the HFD group than in the NC group (*p* < 0.05). The SB group showed a lower percentage of CD68^+^ Kupffer cells compared to the HFD group, but not significantly (20.33 ± 3.42% vs. 30.40 ± 4.90%) (Figure 3D). The proportion of cytokine-producing CD11b^+^ Kupffer cells was significantly higher in the HFD group than in the NC group (*p* < 0.05), and was significantly lower in the SB group than in the HFD group (12.03 ± 1.47% vs. 25.88 ± 4.57%, *p* < 0.05) (Figure 3F).

### 2.4. Effects of SB on Inflammatory Gene Expression

To investigate the mechanism of SB action on inflammatory cytokines and macrophages, we evaluated TNF-α, IFN-γ, and F4/80 mRNA expression levels in liver tissue. The TNF-α expression level significantly increased in the HFD group compared to the NC group (*p* < 0.001), and significantly decreased in the SB group compared to the HFD group (*p* < 0.01) (Figure 3G). The F4/80 mRNA expression level was significantly higher in the HFD group than in the NC group (*p* < 0.001), and was significantly lower in the SB group compared to the HFD group (*p* < 0.01) (Figure 3). However, IFN-γ was similar across groups (Figure 3G).

## 3. Discussion

In an obese state, excessive accumulation of lipids in adipose tissue and liver activates inflammatory cytokine and chemokine secretion, which causes chronic low-level inflammation and insulin resistance. Therefore, obesity becomes a risk factor for many diseases, including metabolic syndrome, cardiovascular disease, and cancer [4,11,12]. If we can modulate the systemic inflammation and insulin resistance as well as reduce lipid accumulation, it can be expected to lower the risk of metabolic syndrome. In this study, we evaluated the effects of a medicinal herb, *Scutellaria baicalensis*, on insulin resistance and metabolic syndrome in mice. SB decreased lipid accumulation in adipose tissue and liver; improved insulin resistance, glucose and lipid metabolism; and modulated the inflammatory macrophage infiltration and cytokine expression in adipose tissue and liver.

The flavones baicalin, wogonoside and their aglycones baicalein and wogonin are the major bioactives in Scutellaria roots, of which baicalin can reduce HFD-induced body weight gain, circulating cholesterol and free fatty acid levels and systemic inflammation [13]. Wogonin may have beneficial effects on glucose and lipid metabolism [14], and baicalein protects mice from metabolic syndrome [15].

In the setting of insulin resistance, a major feature associated with lipid metabolism is an increase in free fatty acids (FFA). An increased FFA level is caused by resistance to insulin action, which inhibits adipose tissue lipolysis [16], and is accelerated by increases in TG storage and adipose tissue [17]. If FFA influx into the liver increases, the production of TG and glucose in the liver increases, and excessive TG is secreted in the form of very low density lipoprotein (VLDL) [18]. Increased VLDL directly contributes to the development of dyslipidemia related to insulin resistance [19]. The characteristics of abnormal lipid metabolism caused by insulin resistance are themselves factors that exacerbate insulin resistance. Lipid accumulation in liver and adipose tissue accelerates insulin resistance by increasing the secretion of pro-inflammatory cytokines [18]. Several studies have reported the effects of SB extract on lipid metabolism. Song [9] reported that SB administration alleviated obesity and hypertriglyceridemia in db/db mice, and Matsuo [20] reported that SB decreased pancreatic lipase activity in vitro and decreased serum TG levels in intralipid-fed ddY mice. Chen [10] reported that SB administration in KK-A^y^ mice reduced serum TG, TC, and FFA levels and improved hepatic TG and hepatomegaly. In this study, SB decreased serum TG and TC levels and improved liver fat accumulation, which was similar to previous studies.

SB showed good effects on the histological characteristics of liver and adipose tissue. SB significantly reduced the weight of both epididymal fat pad and liver, the adipocyte size of adipose tissue, and liver fat area. Epididymal fat is a representative visceral fat widely used in metabolic studies using mouse models [21]. Visceral fat, compared to subcutaneous fat, is deeply involved in metabolic processes because of its active secretion of cytokines and chemokines. Therefore, it is also closely related to insulin resistance [12,22]. In this study, there was a tendency to decrease in body weight with SB administration, but no significant difference was found. This is in contrast to Song’s study [9], in which weight loss was reported as an effect of SB. There are three possible causes for this difference. First, the mouse models were different. Song used ob/ob mice, which have a leptin receptor mutation. Thus, despite the lower dose and shorter dosing period (100 mg/kg body weight for 4 weeks) than in the present study, a significant difference was apparent. Second, OGTT and OFTT performed at week 14–15 may have affected the weight gain patterns. Third, the duration of the experiment may not have been long enough for the weight loss with SB to become apparent. There were two studies in which an HFD murine model was treated with major SB flavonoids. A 16-week baicalin treatment was associated with significant weight loss [13], but a 9-week baicalein administration was not [23]. These results suggest that weight loss occurs if the duration of SB administration is sufficient. However, the effect of SB on insulin resistance was more clearly confirmed in that hyperglycemia, hypertriglyceridemia, and the degree of fat deposition in the tissue improved without significant weight loss.

ATM, which is deeply involved in inflammatory responses, is categorized into two phenotypes: M1 macrophages, marked with CD11c^+^, and M2 macrophages, marked with CD206^+^. M1 macrophages stimulate the inflammatory response through producing pro-inflammatory cytokines. M2 macrophages exhibit anti-inflammatory activities such as inhibition of inflammatory cytokines, and increase insulin sensitivity [24]. Larger and expanded adipose tissue seems to play a major role in the development of insulin resistance [2,25]. Hypertrophic adipocytes are positively correlated with a larger number of pro-inflammatory cytokines [26], which not only directly interfere with the insulin signaling pathway, but also stimulate monocytes to aggregate into adipose tissue and differentiate into macrophages. Activated macrophages accelerate systemic inflammation by secreting their own pro-inflammatory mediators, which leads to the development of insulin resistance [27]. In this study, we showed that SB significantly reduced the proportion of ATMs and significantly increased the proportion of M2 macrophages. Therefore, it seems reasonable that SB suppressed inflammation through modulation of the proportion of ATMs.

Kupffer cells, in other words liver macrophages, are mainly classified into two subsets by surface marker and function. The first is the F4/80^+^ CD68^+^ subset, with high phagocytic activity and reactive oxygen species production. The second is the F4/80^+^ CD11b^+^ subset, which is involved in the inflammatory response due to its high cytokine-producing activity [28]. The activation of Kupffer cells and the resulting hepatic inflammation were found to be key contributors to the development of insulin resistance independent of the effects of lipid accumulation [29]. To investigate the effect of SB on Kupffer cells, especially CD11b^+^ cytokine-producing Kupffer cells, FACS analysis was performed. SB significantly lowered the proportion of CD11b^+^ Kupffer cells. The proportions of total Kupffer cells and CD68^+^ Kupffer cells decreased but not significantly. Therefore, SB appears to suppress inflammation by decreasing inflammatory cytokine-producing CD11b^+^ Kupffer cells.

To further investigate the mechanism of inflammation suppression, analyses of TNF-α, IFN-γ, and F4/80 mRNA expression in liver tissue were performed. SB suppressed the expression of TNF-α and F4/80. TNF-α is a major inflammatory marker, and F4/80 is a marker specific to murine macrophages. IFN-γ activates immune cells, including macrophages, and causes monocytes to differentiate into M1 macrophages [30]. Contrarily, recent study suggest that Interferon-alpha 2, but not Interferon-gamma, are associated with intramuscular fat deposition in obese patients [31]. In this study, unlike other genes, IFN-γ expression did not significantly change in either the HFD or the SB groups. In addition, the fact that SB did not affect the proportion of M1 macrophages suggests that SB does not act on IFN-γ.

Taken together, we have found that SB has favorable effects on hyperglycemia, glucose tolerance, hyperinsulinemia, hypertriglyceridemia, and the histopathological findings of epididymal fat pad and liver. These results suggest that SB can improve insulin resistance through inhibition of macrophage-mediated inflammation. This finding suggests that SB has potential as a therapeutic agent for insulin resistance and metabolic syndrome, and further study including clinical trial should be required to confirm these results.

## 4. Materials and Methods

### 4.1. Preparation of Scutellaria baicalensis (SB)

The root of SB which was harvested from September to October 2017 in Shanxi province, North China, was purchased from the Department of Pharmaceutical Preparation of the Hospital of Korean Medicine, Kyung Hee University (Seoul, South Korea). The quality of the SB was tested according to the standards of the Korea Food & Drug Administration and of our hospital. A voucher specimen (Code number. HHGE/201804) was deposited in our department. One thousand grams of dried SB was boiled with 1500 mL of 80% ethanol using a heating mantle for 2 hours. The extract was transferred to a 500 mL flask by an applicator, and filtered. The filtrate was concentrated with a rotary evaporator (model NE-1, EYELA Co., Tokyo, Japan). The extract was freeze dried and stored at room temperature. The final extraction yield of SB was 41%.

### 4.2. Animals and Treatments

Six-week-old male C57BL/6 mice weighing 19–21 g were purchased from Central Lab. Animal Inc. (Seoul, Korea). They were housed in a room maintained at 40–70% relative humidity under a 12-hour light-dark cycle and fed diet and water *ad libitum* throughout the study. After a 7-day adaptation period, the mice were randomly assigned to one of three groups: normal chow (NC, *n* = 6), HFD (*n* = 6), and SB (*n* = 6). All the mice except the NC group were fed HFD (60% energy by fat), which is known to induce obesity [32] for 17 weeks. After it was confirmed that 8 weeks of HFD feeding made a significant difference (*p* < 0.001) in body weight between the NC group and the other groups, SB (500 mg/kg body weight) [33] was orally administered with zonde daily for 9 weeks, while the NC and HFD group received normal saline. All protocols used in this study were performed with the approval (12 April 2018) of the Animal Care Committee at Kyung Hee University (KHMC-IACUC201813).

### 4.3. Oral Glucose Tolerance Test (OGTT)

At week 14, each mouse was fasted for 14 h and the fasting glucose level (0 min) was measured from a drop of tail vein blood, using a strip-operated blood glucose sensor (Accu-Chek Performa, Australia). Then, each mouse was administered oral glucose (2 g/kg body weight) dissolved in distilled water, and glucose measurements were done from tail vein blood at 30, 60, 120, and 180 min after glucose administration. The glucose area under the curve (AUC) in the OGTT was calculated from measurements taken before (0 min) and after (up to 180 min) glucose administration using the trapezoidal rule, which is a numerical integration method used to approximate the integral or the AUC.

### 4.4. Oral Fat Tolerance Test (OFTT)

At week 15, the fasting triglyceride (TG) level (0 min) was measured from tail vein blood after 14-hour fasting. Then, olive oil (2 mL/kg body weight) was administered orally and TG measurements were taken from tail vein blood at 120, 180, 240, and 360 min after fat administration. The TG measurements were performed by Accutrend Plus (Roche, Brighton, MA, USA). The TG AUC in the OFTT was calculated from measurements taken before (0 min) and after (up to 360 min) fat administration.

### 4.5. Biochemical Assays

At week 15, for insulin concentration measurement, blood was obtained from the tail vein of each mouse after 6-h fasting. Serum insulin concentration was measured using an ultrasensitive mouse insulin ELISA kit (Crystal Chem Inc., Elk Grove Village, IL, USA). From fasting blood glucose and insulin concentration, insulin resistance was assessed using the homeostatic model assessment of insulin resistance (HOMA-IR). At the end of the experiment, after 14-h fasting, serum total cholesterol (TC), low density lipoprotein (LDL) cholesterol, high density lipoprotein (HDL) cholesterol, and TG levels were measured.

### 4.6. RNA Extraction and Analysis of Gene Expression

At week 17, the mice were sacrificed and livers were dissected. RNA extraction was performed using a Mini RNA Isolation IITM (Zymo Research Corp, Irvine, CA, USA). RNA was extracted using TRIzol reagent. To evaluate gene expression including TNF-α, interferon gamma (IFN-γ), and F4/80, quantitative real time-polymerase chain reaction (qRT-PCR) was performed. Prior to qRT-PCR, the complementary DNA (cDNA) was synthesized using an Advantage RT for PCR Kit (Clontech, Mountain View, CA, USA). To the cDNA obtained through reverse transcription PCR, 2× SYBR Reaction buffer, primers, and dH_2_O were added, and qRT-PCR was carried out using 7900HT Fast Real-Time PCR System (Applied Biosystems^®^, Waltham, MA, USA). For gene expression analysis, the threshold cycle for each gene, obtained with SDS Software 2.4 (Applied Biosystems^®^, Waltham, MA, USA), was converted to relative quantitation based on GAPDH, and the fold change was calculated. The fold change value of each experimental group was normalized according to the NC group, which was defined as 1.

### 4.7. Isolation of Stromal Vascular Cells (SVCs) and Liver Immune Cells

At week 17, harvested epididymal fat pads were put into a solution composed of phosphate buffered saline (PBS, Gibco, Waltham, MA, USA) and 2% bovine serum albumin (BSA, Gibco, USA) and minced into 1–2 mm size pieces with round shape scissors. After adding collagenase (Sigma, St. Louis, MO, USA) and DNase I (Roche, USA), a 100-μm cell strainer (BD Biosciences, USA) was used to remove extraneous tissue. The liver was perfused with PBS (pH 7.0) through a needle inserted into the portal vein, and then liver tissue was placed in a 60-mm petri dish with RPMI 1640 medium containing 100 mL/L fetal calf serum (FCS) and pulverized into small pieces. The sample was filtered through a 200-gauge stainless mesh and mixed with 9 mL of PBS, 8 mL of Percoll (final 36.3%), and then 200 μL heparin, and the mixture was centrifuged at 2000 rpm for 20 min. After removal of the supernatant containing parenchymal cells, 1× ACK lysis buffer (Lonza) was added to the pellet to dissolve the red blood cells. The sample was finally centrifuged at 1500 rpm for 5 min to obtain non-parenchymal cells containing immune cells collected in the lower layer.

### 4.8. Fluorescence Activated Cell Sorting (FACS) Analysis of Adipose tissue macrophages (ATMs) and Kupffer Cells

Each sample was prepared to contain 10^6^ cells. A mixture of FcBlock (BD Pharmingen, San Jose, CA, USA) and fluorophore-conjugated antibodies was added to each sample. The antibodies used for the analysis of ATMs were CD45-APC Cy7 (Biolgend, San Diego, CA, USA), CD68-APC (Biolgend, USA), CD11c-phycoerythrin (CD11c-PE, Biolgend, USA), and CD206-FITC (Biolgend, USA); and for liver Kupffer cell analysis were CD45-FITC (Biolgend, USA), F4/80-APC (Biolgend, USA), CD68-PE (Biolgend, USA), and CD11b-PerCp CY5.5 (Biolgend, USA). After washing with 2% FBS/PBS solution, each sample was centrifuged at 1500 rpm and transferred into a fluorescence activated cell sorting (FACS) tube. The analysis was conducted with FACS Calibur (BD Bioscience, USA). The percentages of ATMs with CD45^+^ CD68^+^, CD45^+^ CD68^+^ CD206^+^, and CD45^+^ CD68^+^ CD11c^+^ and the percentages of Kupffer cells with CD45^+^ F4/80^+^, CD45^+^ F4/80^+^ CD68^+^, and CD45^+^ F4/80^+^ CD11b^+^ were analyzed using FlowJo (Tree Star, Inc., Ashland, OR, USA).

### 4.9. Histological Analyses of Adipose Tissue and Liver

Obtained epididymal fat pad and liver samples were fixed in 10% neutral buffered formalin, and embedded in paraffin to make paraffin blocks. Each block was sliced into 4-μm-thick sections with a microtome and attached to a gelatin coated slide. Two sections per animal were stained with hematoxylin and eosin, and digital images were obtained using a high-resolution camera-mounted optical microscope (Olympus BX-50, Olympus Optical, Tokyo, Japan) connected to a computer. Using ImageJ, the adipocyte size in fat tissue and the fat area in liver tissue were measured.

### 4.10. Statistical Analysis

Statistical analysis was performed by GraphPad PRISM 6 (GraphPad software Inc., San Diego, CA, USA). One-way analysis of variance was used to test the statistical differences between the groups and it was followed by Tukey’s HSD test. All values were presented as mean ± standard error. A two-tailed *p* value of < 0.05 was considered statistically significant.
